# Four-Phase, Definitive Chemoradiation for a Real-World (Poor Risk and/or Elderly) Patient Population With Locally Advanced Non-small Cell Lung Cancer

**DOI:** 10.7759/cureus.29423

**Published:** 2022-09-21

**Authors:** Yu M Zhou, Jacob Shin, Michael Jelinek, Mary J Fidler, Marta Batus, Philip D. Bonomi, Gaurav Marwaha

**Affiliations:** 1 Radiation Oncology, Rush University Medical Center, Chicago, USA; 2 Radiation Oncology, Memorial Sloan Kettering Cancer Center, New York City, USA; 3 Medical Oncology, Rush University Medical Center, Chicago, USA

**Keywords:** intensity modulated radiotherapy, concurrent chemoradiation therapy, advanced non-small-cell lung cancer, geriatric medicine, palliative radiation therapy

## Abstract

Introduction

With the incorporation of modernized radiotherapy, chemotherapy, and immunotherapy, treatment outcomes have improved for patients with locally advanced, unresectable diseases. Elderly or poor performance status patients comprise more than half of non-small cell lung cancer (NSCLC) patients, but they are often underrepresented or excluded in clinical trials. Split-course concurrent chemoradiotherapy can be an effective treatment, showing good adherence and a favorable toxicity profile for unresectable, locally advanced NSCLC.

Method

We identified locally advanced NSCLC cancer patients via a single institution retrospective study. Patients were treated using a four-phase, split-course external beam radiotherapy approach with concurrent chemotherapy. The primary endpoints analyzed were completion rate, incidence, and severity of treatment-related toxicities, progression-free survival (PFS), and median overall survival (OS).

Results

Thirty-nine locally advanced lung cancer patients were treated with split-course chemoradiation (CRT). The median age at diagnosis was 73 years old. Seventeen patients had an Eastern Cooperative Oncology Group (ECOG) performance score of 2. Twenty-three patients had a clinical diagnosis of chronic obstructive pulmonary disease (COPD), and 10 patients were on home oxygen at the time of diagnosis. All patients completed 6000 centigrays (cGy) of radiation, and 95% of the patients completed at least three cycles of concurrent chemotherapy. No patients experienced grade 3 to 5 acute thoracic toxicities. Overall median survival was 12.7 months, and PFS was 7.5 months.

Conclusion

Our retrospective analysis of 39 poor risk and/or elderly patients with locoregional NSCLC treated with concurrent CRT via a split-course regimen suggests favorable oncologic outcomes and superb treatment completion rates and toleration.

## Introduction

Lung cancer is the number one cause of death among all malignancies, with an estimated 135,720 deaths in the year 2020. Non-small cell lung cancers (NSCLC) make up most primary lung cancers and approximately 70% of lung cancer patients initially present with locally advanced or metastatic disease [1]. With the incorporation of chemotherapy, modernized radiotherapy delivery techniques, and more recently immunotherapy, locoregional tumor control and overall survival (OS) rates have improved for patients with locally advanced, unresectable diseases [2-6]. While landmark clinical trials have shown that outcomes have improved over time, a significant proportion of patients do not meet the trials' inclusion criteria. In the two most recent landmark trials, both RTOG 06-17 and PACIFIC excluded patients with performance scores of two or higher. Furthermore, the median age of the patients in both trials was 64 years old [5,7]. According to the Surveillance, Epidemiology, and End Results (SEER) database, 70% of newly diagnosed lung cancer are 65 years old or older, and the median age of diagnosis is 71 years old [8]. Elderly or poor performance status patients comprise more than half of NSCLC patients, but they are often underrepresented or excluded in clinical trials [9,10]. Unfortunately, these patients have poorer survival, even with the inclusion of immunotherapy [11]. The data show that the actual patient population may be quite different than the patients enrolled in the recent clinical trials. Standard concurrent chemoradiation (CRT) for stage III NSCLC is a six-to-seven week continuous course of therapy, with consolidative immunotherapy afterward. Unfortunately, there are significant adverse events with the standard of care CRT, often resulting in the discontinuation of therapy. According to various studies, the discontinuation rate of concurrent CRT can be as high as 30% [12,13]. As a result, patients with poor performance status and/or elderly patients are often treated with more palliative treatments. With the risk of increased morbidity and/or therapy discontinuation in elderly and poor performance status patients, clinical studies are needed to develop more feasible and less toxic options. Split-course concurrent chemoradiotherapy has demonstrated effectiveness, good adherence, and a favorable toxicity profile in both the definitive and palliative contexts for unresectable, locally advanced NSCLC [14,15]. Furthermore, in a randomized trial evaluating split-course radiation and simultaneous daily cisplatin versus radiation alone, the chemotherapy/radiation treatment arm was associated with superior survival [16]. More recently, a prospective phase II clinical trial showed minimal grade three toxicity with promising survival for medically fit patients treated with split-course radiation [17]. In this retrospective study, we aim to assess the tolerability and estimate OS in real-world patients treated with split-course thoracic radiation and concurrent chemoradiotherapy using modern radiation planning techniques in the context of poor performance status and/or elderly patients with locoregional locally advanced NSCLC.

## Materials and methods

Patients

Institutional Review Board approval was obtained for this retrospective study. We identified 126 locally advanced lung cancer patients via patient database chart review. The Eastern Cooperative Oncology Group (ECOG) performance scale was used as the assessment tool for functional impairment, where poor risk was defined as an ECOG performance score of two or greater. All patients were stages II to III according to the American Joint Committee on Cancer (AJCC) Manual, eighth edition [18]. Patients were treated using a four-phase, split-course external beam radiotherapy approach with concurrent CRT. Patients were prospectively evaluated and managed via a multidisciplinary clinic with participating radiation oncologists, medical oncologists, and thoracic surgeons.

Treatment regimen

**Figure 1:**
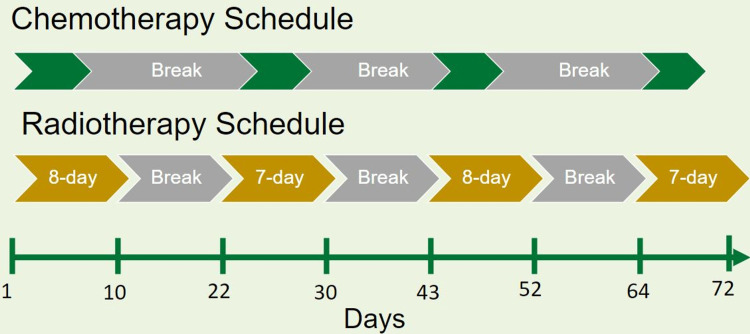
Four-phase split-course chemotherapy and radiation schedule.

Split-course CRT consisted of four 21-day treatment cycles. Radiation was delivered on seven or eight of the first 10 weekdays of each cycle, and platinum-doublet regimens started on days one to three of each radiation cycle. The last week of each cycle was a break for all therapies. Figure 1 shows the chemotherapy and radiation treatment schedule. All patients were treated with 6 megavolts (MV) photons alone or with a mixture of 10 or 18 MV photons from a linear accelerator using three-dimensional conformal radiotherapy (3D-CRT) or intensity-modulated radiotherapy (IMRT) in daily fractions of 200 cGy.

At the time of radiation, the treatment field was designed to encompass the detected tumor and involved nodal stations on CT and/or positron emission tomography (PET) scans. No elective nodal irradiation was given. The motion of the lesions observed under four-dimensional (4D) planning CT was considered and included in the internal target volume (ITV). A clinical target volume (CTV) margin of 5 mm was added to the ITV for primary lung tumor, and an ITV margin was added to the involved nodal CTV station. Composite ITV+CTV for primary lung tumor and nodal volumes was expanded by 5 mm to the planning target volume (PTV) to accommodate setup uncertainty. Volumes were consistent throughout all four phases with no re-planning. Dose constraints were based on RTOG 0617 protocol. Major constraints included lung V20 (volume receiving 20 Gy), limited to <30%, heart mean dose <26Gy, and esophagus mean dose of <34Gy. Chemotherapy was delivered in the form of a systemically dosed platinum-doublet. Patients generally received carboplatin area under the curve of 4 or 5 every 21 days or cisplatin 60 mg/m2 every 21 days, with either pemetrexed 500 mg/m2 on day 1, etoposide 80 mg/m2 on days one to three, or paclitaxel 80-100 mg/m2 on days one and eight.

Outcomes

The primary endpoints analyzed were completion rate, incidence and severity of treatment-related toxicity, progression-free survival (PFS), and median OS. Completion was defined as the completion of 6000 cGy of radiation and at least three cycles of chemotherapy. Treatment-related toxicities were assessed according to the Common Toxicity Criteria for Adverse Events version 5.0 [19]. PFS was defined as the time between the start of split-course CRT and the date of disease progression, either locally or distantly, or the last follow-up. Disease progression was determined on imaging by Response Evaluation Criteria In Solid Tumors (RECIST version 1.1) criteria using follow-up chest CT and/or PET scan [20]. Disease progression was deemed to have occurred when the progressive disease was demonstrated on follow-up CT and/or PET scans and documented on the radiologist report and/or biopsy-proven. Median survival was defined as the length of time from the start of split-course CRT to when half of the patients diagnosed were still alive.

## Results

Patient and treatment characteristics

We identified 39 locally advanced lung cancer patients consecutively treated with split-course CRT between 2015 and 2020. The patient and treatment characteristics are shown in Table 1. A total of 22 patients were female (56.4%), and the median age of diagnosis was 73 years old (range: 47-83 years old). Seventeen patients had an ECOG performance score of 2 (43.6%), and there were no patients with a score of three or higher. A total of 22 patients had a clinical diagnosis of chronic obstructive pulmonary disease (COPD) (59.0%), and 10 patients were on supplemental oxygen at the time of diagnosis (25.6%). Thirty-eight patients had biopsy-proven locally advanced lung cancer. Nineteen had squamous cell carcinoma, 15 adenocarcinomas, and four had poorly differentiated carcinomas. All patients completed 6000 cGy of radiation prescribed with a 95% isodose line, and 95% of the patients completed at least three cycles of concurrent chemotherapy. In terms of radiation planning, 82.1% of patients were treated with volumetric arc therapy (VMAT); 10.3% of patients were treated with static intensity-modulated radiotherapy (IMRT); 7.7% of patients were treated with three-dimensional conformal arc therapy. Average lung V20 was 24%; heart mean dose was 14 Gy; and esophagus mean dose was 21 Gy. Most patients received carboplatin and etoposide (n=20, 51.3%), and 11 received consolidative immunotherapy (28.2%). Of the 13 patients receiving adjuvant immunotherapy, nine received durvalumab, one received nivolumab with ipilimumab, and one received atezolizumab. In addition, eight patients received salvage immunotherapy at the time of progression (20.5%). The final follow-up was performed on January 12, 2021, and the follow-up period from the start of split-course CRT was 18.3 months.

**Table 1:** Clinical and treatment characteristics.

Characteristics	Number (%)
Gender No. (%)	
Female	22 (56)
Male	17 (44)
Age at Diagnosis (years)	
Median (range)	73 years (47-83)
ECOG Performance Score No. (%)	
0-1	22 (56)
2	17 (44)
Disease Stage No. (%)	
IIIA	15 (38)
IIIB	19 (49)
IIIC	5 (13)
Tumor Histology No. (%)	
Adenocarcinoma	15 (38)
Squamous cell carcinoma	19 (49)
Poorly differentiated	4 (10)
History of COPD No. (%)	23 (59)
Home oxygen No. (%)	10 (26)
Average doses to organs at risk	
Lung V20	24%
Heart mean	14 Gy
Esophagus	21 Gy
Chemotherapy Regimen No. (%)	
Carboplatin/Etoposide	20 (51)
Carboplatin/Pemetrexed	14 (36)
Carboplatin/Paclitaxel	3 (7)
Cisplatin/Etoposide	1 (3)
Etoposide	1 (3)
Adjuvant Immunotherapy Regimen No. (%)	
Durvalumab	9 (23)
Nivolumab and Ipilimumab	1 (3)
Atezolizumab	1 (3)

Acute toxicity

The incidence of acute toxicity by grade according to Common Terminology Criteria for Adverse Events, Version 5.0 (CTCAE v5.0) is shown in Table 2. The most commonly reported acute toxicity was grade one fatigue in 30 patients (77%). Five patients (13%) experienced grade one radiation pneumonitis, and two (5%) experienced grade two radiation pneumonitis. Seven (18%) had a grade one dysphagia, while three patients (8%) experienced grade two dysphagia. No patients experienced grade three to five acute thoracic toxicities. There were no episodes of neutropenic fever, and there were no grade five hematologic toxicities.

**Table 2:** Acute toxicity by grade (Common Terminology Criteria for Adverse Events, Version 5.0).

	0	1	2	3	4	5
Dysphagia	29 (74%)	7 (18%)	3 (8%)	0	0	0
Pneumonitis	32 (82%)	5 (13%)	2 (5%)	0	0	0
Dyspnea	26 (67%)	11 (28%)	2 (5%)	0	0	0
Dermatitis	29 (74%)	8 (21%)	2 (5%)	0	0	0
Fatigue	9 (23%)	30 (77%)	0	0	0	0
Nausea	38 (97%)	1 (3%)	0	0	0	0
Weight loss	32 (82%)	7 (18%)	0	0	0	0

Outcomes

The median follow-up of all patients was 18.3 months. There was a 100% completion rate for patients treated with split-course radiation, and 95% of the patients completed concurrent CRT. 22 patients had a partial response at the time of three months follow-up. There were no complete responders. Overall median survival was 12.7 months, and PFS was 7.5 months.

## Discussion

Our study is the first to apply split course CRT specifically to a poor-risk patient population in a definitive manner. Considerable caution must be exercised when planning treatment for a patient group with much poorer outcomes using standard concurrent CRT. As such, the potential benefits of aggressive locoregional therapy must be carefully weighed against the increased likelihood of adverse side effects in those with poor risks. This study showed an inordinately low toxicity profile (zero grade 3+ toxicities) and a 95% completion rate of concurrent CRT. In addition to improved tolerability, our patient cohort had a low rate of adverse events, which commonly occur in the overall population of advanced lung cancer patients [21]. The 95% completion rate and minimal radiation or CRT delays also contributed to the good outcome in our patients. As discussed earlier, these patients often are not offered curative therapy options. With split-course CRT, employing highly conformal radiotherapeutic techniques and interdigitated treatment breaks, we can offer patients with poor risk a more durable palliative therapy while minimizing side effects and discontinuation.

The advanced age and poor performance status significantly impact treatment outcomes. In a retrospective review of Medicare patients with locally advanced NSCLC from the SEER database, only 66% of the patients older than 65 years old received any cancer treatments, and only 45% of those treated received a standard approach of combined chemotherapy and radiation [22]. Elderly patients and/or patients with poor performance status who received concurrent chemoradiation often have poor survival outcomes. A post hoc analysis of cancer and leukemia group B (CALGB) trial 39801 identified four factors that were predictive of decreased survival: 1) age greater than or equal to 70 years; 2) performance score of 1; 3) weight loss greater than or equal to 5%; and 4) hemoglobin level less than 13g/dL. The median OS for patients was 18 months versus nine months in patients with two or more of these factors [23]. Not only do these patients have worse survival outcomes, but they also have significantly increased toxicities during treatment. One of the more common side effects of concurrent CRT is esophagitis. In the previously discussed meta-analysis by the Non-small Cell Lung Cancer Collaborative Group, concurrent CRT had significant grade three to four esophageal toxicities (18% vs. 4%) compared to sequential CRT. Studies have shown that concurrent chemoradiation toxicities increase six-fold in elderly patients with stage III NSCLC [24]. However, a search of active clinical trials reveals a lack of practice-changing trials for the elderly [25].

Our outcomes were similar to historically published studies of split-course CRT [14-16]. In a single-institutional retrospective study on 144 medically fit patients with locally advanced NSCLC, definitive split-course CRT was delivered consisting of four treatment cycles, each cycle 21 days in length using a cumulative dose of 6000-6400 cGy concurrent with systemically dosed platinum-doublet CRT [14]. Grade three or higher esophagitis was uncommon, with a crude rate of 3%, and the crude rate of grade three to four pneumonitis was 14.5%. The median OS was 20.4 months. In a separate single-institutional retrospective study of 55 patients with incurable locally advanced or metastatic NSCLC, palliative split-course CRT was delivered using a median dose of 4000 cGy over 20 fractions concurrent with a systemically dosed platinum-doublet [15]. There were no cases of grade 3 toxicity. The actuarial 6-, 12-, and 24-month cumulative incidence of locoregional failure was 6%, 14%, and 22%, respectively. The studies appear to suggest that split-course radiation therapy with concurrent CRT may allow for durable locoregional control with acceptable morbidity.

Our investigation has several limitations inherent to retrospective analysis, and the reported results need to be interpreted with caution due to the potential of significant selection biases. Our institutional experience represents outcomes in a selected patient cohort, and protracted concurrent chemoradiotherapy may not be appropriate for those with significant medical comorbidities or very-short life expectancy (<three months). Others have questioned the merit of a split-course approach in an era of significant advances in radiotherapy technology and delivery, and the development of molecular targeted agents which may preferentially sensitize tumors [26]. The treatment breaks in split-course can be viewed as interruptions in delaying the completion of planned radiation therapy. This may be associated with poorer tumor control rates and patient prognosis [27,28]. However, since most patients in the population often receive palliative doses of radiation, split-course offers near more durable treatment for carefully selected patients. Rarely are elderly poor-performance patients offered concurrent CRT, which has been the foundation of inoperable advanced NSCLC. Our split-course CRT regimen was able to better provide appropriately selected patients with aggressive, definitive-type treatment for their locoregional disease while giving them the best chance of completing their treatment course without unscheduled interruptions/hospitalizations or significant toxicity.

## Conclusions

Our retrospective analysis of 39 poor risk and/or elderly patients with locoregional NSCLC treated with split-course chest radiation, and concurrent platinum CRT shows a high rate of treatment completion and encourages OS. This treatment strategy appears to be an appropriate option for high-risk stage III NSCLC patients. This study is the basis for an ongoing phase II trial evaluating split-course chest radiation and concurrent platinum-doublet CRT followed by durvalumab.
